# Association of Polymorphic and Haplotype Variants of the *MSX1* Gene and the Impacted Teeth Phenomenon

**DOI:** 10.3390/genes12040577

**Published:** 2021-04-16

**Authors:** Grzegorz Trybek, Aleksandra Jaroń, Anna Grzywacz

**Affiliations:** 1 Department of Oral Surgery, Pomeranian Medical University in Szczecin, Powstańców Wielkopolskich 72/18, 70-111 Szczecin, Poland; aleksandra.jaron@pum.edu.pl; 2 Independent Laboratory of Health Promotion, Pomeranian Medical University in Szczecin, 70-204 Szczecin, Poland; grzywacz.anna.m@gmail.com

**Keywords:** *MSX1*, genetic polymorphism, association studies, impacted teeth, odontogenesis

## Abstract

It is known that genetic factors determine odontogenesis; furthermore, studies have revealed that various genes in humans can regulate the development of different types and generations of teeth. In this study it has been assumed that tooth impaction—at least to some extent—also depends on the presence of specific genetic markers, especially allelic variants of the *MSX1* gene. The primary objective of the study was to evaluate the suitability of selected molecular markers located within the *MSX1* gene for the determination of the risk of tooth impaction in particular patients. The study participants were divided into two groups: (1) the study group—at least one secondary tooth was impacted in the jaws; (2) the control group—no impacted tooth in the jaws. Real-Time PCR and TaqMan probes were used to detect selected polymorphisms in the analyzed genes. Two single nucleotide polymorphisms of *MSX1* were analyzed. After the two subgroups of patients were distinguished in the study group based on the number of impacted teeth, statistically significant differences in the frequency of genotypes described for *rs12532* in the *MSX1* gene were found.

## 1. Introduction

Molecular pathways and morphogenetic processes, which are involved in the development of teeth, have been studied thoroughly over the last few decades. Mice are the most popular animal models used in this type of research. Generally, the tissues necessary for the formation of proper dentition come from two sources. The epithelial tissue comes from the superficial ectoderm and the pharyngeal endoderm [1,2], whereas mesenchyme is formed by the cells of the cranial neural crest. The neural crest cells originate from the marginal part of neuroepithelium and migrate laterally and ventrally to fill facial protuberances with mesenchyme [3]. Mesenchyme, which comes from the neural crest, ultimately forms the skeletal elements of the face and maxilla, as well as most of the soft and hard tissues in teeth, including dentin, dental pulp, alveolar process bone, and periodontal ligaments [4].

In this day and age, genetic control of odontogenic processes raise no doubts. It is a well-known fact that genetic factors determine tooth development. What is more, it has been found that various genes may control the development of various types and generations of teeth in vertebrates. The research studies carried out over the past few years on mouse embryos allowed to identify over 300 genes whose protein products are involved in tooth development [5]. First, it was shown that the process of odontogenesis, like other developmental processes, is strictly controlled by homeobox genes (*HOX*), which code for numerous factors that regulate the differentiation of mesenchymal cells and their receptors, and as previously mentioned, embryological studies confirmed that induction of the process of tooth formation requires that the two types of tissues—epithelium and neural mesenchyme—interact. It was also confirmed that apart from the genes belonging directly to the *HOX* family, other collaborating genes that are involved in controlling odontogenesis also exist and code for factors responsible for signaling interactions between epithelium and mesenchyme during tooth development, determining the position, type, size, and shape of teeth [6]. Many of these genes are also involved in the development of other tissues, which explains the occurrence of dental defects in numerous syndromes, in conjunction with abnormalities in other organs. The formation of subsequent developmental stages, leading to the development of a mature tooth of a correct structure, is controlled by numerous genes regulating other genes’ expression. Their products influence transcription processes which are important for the course of developmental transformations. Observations of odontogenesis, which have been carried out to date, indicate that this process is controlled by numerous signaling pathways which have their impact on the response to both intercellular interaction signals and signals generated by the environment, and are also responsible for controlling cellular cycles. It was found that *TNF* (tumor necrosis factor) signaling pathways, *FGF* (fibroblast growth factor) signaling pathways, BMP (bone morphogenetic protein) signaling pathways, *SHH* (sonic hedgehog) signaling pathways, and WNT (wingless-related integration site) signaling pathways are activated in a coordinated manner during organogenesis, and are involved, among others, in the initiation of tooth development. Any mutations in the genes encoding factors in the above-mentioned signaling pathways, which have their impact on the disordered functioning of molecules encoded by them, may result in an incorrect genesis, development as well as eruption and functioning of teeth. Numerous components are involved in all the above processes in which transcription factors that interact directly with DNA—and in this manner control expression of particular regions in genetic material containing genes that are crucial for tooth development—are particularly important [7].

It is most often that impacted teeth are permanent canines and incisors in the jaw as well as third molars and second premolars in the mandible. According to various authors, impacted teeth in the anterior region are found in 0.9–4.3% of the population (including 70% of canines and 11.5% of incisors). However, the diagnosis of impaction in relation to the lower third molars varies depending on the population under study and the period in which the study is carried out. For the population studied in the 1960s, it ranged from 17% [8,9] to over 70% of young Europeans examined in the early 2000s [10].

The primary reasons for impaction include insufficient space in the dental arch and/or unfavorable angulation, which normally results from a disproportion between the development of a tooth and jaw or mandible development. It is third molars that are most often impacted, since they are the last ones that are through and usually have insufficient space in which they can erupt. Central jaw teeth also get impacted as a result of other teeth crowding [11]. The reasons for tooth impaction may be looked at in more detail as a consequence of unfavorable position of the tooth primordium resulting from its inversion, atypical development of the impacted tooth, a few mutually blocked teeth which are impacted, injury received by the region of the alveolar process (most often in the anterior part) within the period of deciduous teeth, presence of supernumerary or additional teeth as well as odontoma or odontogenic cysts, incorrect resorption of homonymous deciduous teeth roots, occurrence of persistent deciduous teeth, bone scars, gingival fibromatosis, homonymous deciduous teeth re-inclusion, and tooth ankylosis. Tooth impaction may occur with other defects, including cleft alveolar process and/or palate. Endocrine disorders, vitamin D deficiency, and hereditary factors are listed as systemic reasons [12].

The above information shows that the etiology of abnormalities in tooth development is related to the co-occurrence of inherent genetic components and specific environmental conditions under which the very odontogenesis and development of the whole body takes place. It is also beyond doubt that the molecular basis for tooth defects is complex and polygenic in character; therefore, it is a great challenge to indicate individual genetic factors which are responsible for their occurrence. However, the direct involvement of protein products related to the genes including *MSX1* in the processes of odontogenesis has been well documented. Hence, these genes may be regarded as promising molecular goals in research studies on genetic determinants of particular dental abnormalities. Numerous original publications and meta-analyses confirm the importance of diversifying these genes in the etiology of numerous developmental disorders of the skull and occlusion, particularly in abnormal development of tooth primordia and various forms of their agenesis or impaction [13]. As previously mentioned, what is recognized as the primary reason for the occurrence of the impacted teeth phenomenon is a disproportion between tooth development and skull (or mandible) growth, which results in an insufficient space for a particular tooth. Hence, there is a suspicion that disorders in the structure and/or functioning of the above genetic factors—which are directly involved in tooth and periodontal tissue development—may be of importance to the emergence of impacted teeth in people who are predisposed to undergo that sort of change in their genetic material. Therefore, one may assume that the impacted teeth phenomenon is also related, at least partially, to the occurrence of specific genetic markers amongst which allelic variants of *MSX1* genes may be statistically significant.

The primary objective of the study was to evaluate the suitability of selected molecular markers located within the *MSX1* gene for the determination of the risk of tooth impaction in particular patients.

It was hypothesized that the evaluated statistical significance will identify specific genetic elements (alleles/haplotypes/genotypes) pre-sent in selected regions of the polymorphic *MSX1* gene with significantly higher or lower frequency in the study group of patients compared to the control group, and these elements could be important for the assessment of the risk of tooth impaction.

## 2. Results

The study group included 204 patients—82 males and 122 females—who were identified with up to seven impacted teeth (median 3, interquartile range 2–4). The control group included 188 patients. Both in the study group and the control group two patients in the process of genetic testing provided the result of genetic testing which was unclear or was not recorded as a result of the absence of signal. Table 1 and Table 2 show the number of impacted teeth in the study group and the Proportion of different tooth types (according to the Viohl system) in the study group.

**Figure 1:**
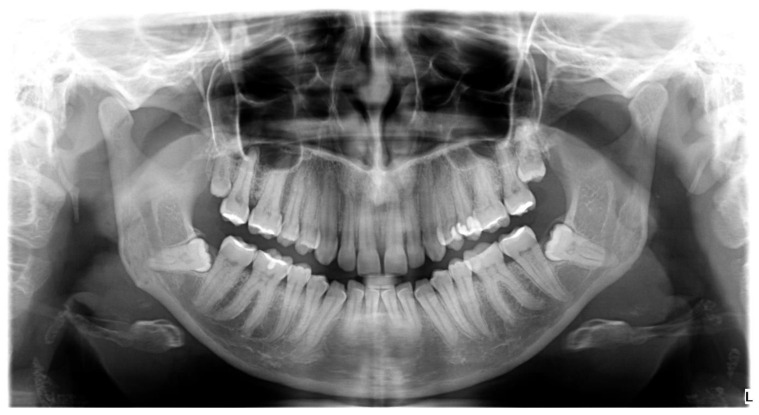
Impaction of four third molars on orthopantomogram (OPG).

Impaction of four teeth was observed in the largest number of patients in the study group, whereas patients with two impacted teeth were the second most numerous subgroup (Figure 1).

A detailed analysis depending on the type and position of impacted teeth and the number of non-erupted teeth is presented in Table 3. It was found that amongst 204 patients in the study group, the most frequent tooth impaction was molar impaction, especially with regard to lower third molars. The biggest number of cases related to lower molars. 75.5% related to tooth 38, and 70.6%—only slightly less frequently—to tooth 48, whereas more than half of the patients under study had impaction of upper molars: 54.4% (tooth 28) and 52.2% (tooth 18). Significantly fewer cases of impaction were recorded for the canines, and they mainly related to the upper canines: 5.4% with impaction of tooth 23 and 2.9% with non-eruption of tooth 13. As for the lower canines, there was only one person in the study group with impacted tooth 43, but no impaction of tooth 33 was observed. As previously mentioned, the biggest number of people in the study group (72) had four teeth simultaneously impacted. Each patient had all molars impacted (18, 28, 38, and 48), except for one, whose tooth 48 was erupted, but tooth 23 was impacted. Amongst patients with two teeth impacted, this condition also affected lower molars: 81.8% (tooth 38) and 74.5% (tooth 48).

The analysis of two polymorphic loci, i.e., *rs8670* and *rs12532* in the *MSX1* gene showed no statistically significant differences in the frequency of genotypes (*p* = 0.487 and *p* = 0.925, respectively) and in the frequency of alleles (*p* = 0.228 and *p* = 0.975, respectively) described at these polymorphic loci in the *MSX1* gene (Table 4 and Table 5).

For all patients qualified for the project haplotype reconstruction based on genotypic data was performed. Three haplotypes of the *MSX1* gene were identified (*rs8670*; *rs12532*): (C; A), (C; G), (T; A) with a total frequency of 100%.

The differences in haplotype frequencies were not statistically significant between the study group and the control group. Irrespective of the assumed haplotypic effect, i.e., additive, dominant, or recessive, both at the level of global tests and the level of tests for significance for particular haplotypes, none of the above-mentioned haplotypes showed any association with belonging to the study group or the control group (Table 6).

When the subgroup of patients with the number of impacted teeth below the median (NIT ≤ MED) was compared to the subgroup of patients with the number of impacted teeth above the median (NIT > MED) with the *rs12532* polymorphic locus in the *MSX1* gene taken into account, statistically significant differences in the frequency of genotypes were found (*p* = 0.026). Moreover, it was shown that the frequency of the G allele described for this locus was 1.6 times lower in the subgroup of patients with a bigger number of impacted teeth (NIT>MED) than in the NIT ≤ MED subgroup (χ^2^ = 6.91, *p* = 0.009, OR = 0.53 [0.33–0.86]) (Table 7). On the other hand, an analogous comparison made for the second of the polymorphic loci in the *MSX1* gene, rs8670, did not reveal any significant differences.

The results of the analysis of the relationship between the *MSX1*gene haplotypes and the number of impacted teeth are presented in Table 8. As for the analysis of haplotypes, at the level of global tests, the relationship with the number of impacted teeth was found with regard to the haplotypes described for the *MSX1*genes. For the *MSX1* gene, statistical significance was found at the assumption of the additive effect (score = 6.44, *p* = 0.040) and the dominant effect (score = 9.65, *p* = 0.022). At the level of individual significance tests, for the three haplotypes described for the *MSX1* gene, the [C; G] haplotype showed association for both the additive model (score = −2.48, *p* = 0.013) and the dominant model (score = −2.64, *p* = 0.008). The frequency of this haplotype was 1.6 times lower in the NIT > MED group than in the NIT ≤ MED group (0.20 vs. 0.32).

## 3. Discussion

The locus of the *MSX1* gene in humans is in chromosome 4. The *MSX1* gene itself consists of two exons—2332 and 1229 bp long, respectively—separated by a 2332 bp long intron. This gene is subject to an alternate reading phenomenon, as a result of which the use of two transcription start sites—located at 236 and 254—generates two distinct transcripts which form the base for two polypeptides: 303 and 297 amino acids long, respectively [14,15]. As mentioned in the introduction, studies using animal models point out that the transcription factor encoded by the *MSX1* gene regulates expression of many target genes and, through direct or indirect interaction, it may interact with PAX9 and other proteins involved in odontogenesis, including the previously discussed protein family, i.e., DLX and TBP [16]. The *MSX1* gene belongs to the group of genes which code for proteins containing the so-called homeodomain that is responsible for binding to specific sequences in DNA of genes regulated by *MSX1* and for interactions with other regulatory proteins. These genes, including *MSX1*, play a key role in odontogenesis, controlling various stages of tooth development and influencing fully developed dentition patterns [17].

Studies using laboratory animals have shown that mice with a blocked *MSX1* gene—as in the case of mice deficient in the *PAX9* gene activity—show a number of abnormalities in the anterior cranial structure, including cleft palate and inhibition of the development of tooth primordia, which reach the maximum bud stage [18]. In such animals, expression of the *BMP4* gene in dental mesenchyme is significantly reduced, which, as a result, causes disturbances in the formation of enamel knots within the dental epithelium region [19]. Studies have shown that the MSX1 protein is one of the key transcription factors involved in the maintenance and regulation of expression of the mesenchymal factor *BMP4*, which is responsible for the progression of tooth morphogenesis in the transition from the bud stage to the cap stage [20,21]. More detailed molecular analyses suggest that interaction occurs both between the *MSX1* and *PAX9* genes, as well as between proteins they code for, and its effect is activation of *BMP4* expression [20,22]. Using a mouse model, it was shown that *MSX1* may also support the function of the ISL1 (ISL1 transcription factor LIM/homeodomain) proteins through the BMP4-dependent pathway and *BARX1* [23,24]. Within this arrangement, PAX9 may regulate *MSX1* expression at the level of the *MSX1* gene as well as through direct interactions with the MSX1 protein, enhancing its effect on the BMP4 protein during odontogenesis [20,22]. DLX proteins may also interact with MSX1 in early stages of tooth morphogenesis [25].

The analysis of the literature reveals that the *MSX1* gene variants are almost as frequently studied for their potential role in the etiology of numerous abnormalities and disorders in tooth development in humans, as are the *PAX9* gene variants. In the early results of some studies it was already suggested that *MSX1* mutations, such as reading frameshift or missense mutations, may be associated with hypodontia [26]. Interestingly, most of the mutations in the *MSX1* gene—which, so far, have been associated with hypodontia in humans—are located in this part of the gene that codes for a highly conservative homeodomain which is responsible for the ability of the transcription factor to bind to specific regions of DNA in target regulatory genes and interact with other regulatory proteins, such as, for example, PAX9. Structural studies have shown that the homeodomain spans 60 amino acids—from residue 166 to 225—that is divided into the N-terminal arm and helices I, II, and III. It was originally assumed that missense mutations in the regions of the *MSX1* gene coding for the homeodomain could disrupt stability of the encoded protein, reduce DNA binding specificity in target genes, reduce expression of the *MSX1* gene itself, as well as the ability of the MSX1 protein to interact with other regulatory proteins.

A functional analysis of the first polymorphism (Arg196Pro)—located in the region of the *MSX1* gene coding for the homeodomain and identified as a genetic factor responsible for the occurrence of hypodontia—revealed that a proline-containing protein has a defective structure and reduced thermal stability, which results in a reduced or completely disabled capacity of binding to DNA and interacting with other proteins. Carrying such a mutation resulted in the more frequent absence of second premolars and third molars, and in some individuals also the upper first premolars and the lower first molars [27]. Studies on a Chinese population identified a new C662A mutation in the coding region of the homeodomain in the *MSX1* gene, which resulted in the Ala221Glu substitution in region III of the homeodomain helix [28]. It is a region of the protein that is not only responsible for binding to DNA, but also for interactions with other proteins, e.g., proteins from the DLX family. It was shown that this mutation may disturb proper functioning of the MSX1 protein through various mechanisms related to the obstruction of formation processes of heterodimeric MSX1 and DLX protein complexes whose task is to initiate epithelial–mesenchymal signal cascades during odontogenesis through functional antagonism [25]. Selective teeth missing, including premolars and third molars, and in some cases lateral incisors and canines, have been found in carriers of the Ala221Glu mutation [28]. Another example of a mutation in the region of the *MSX1* gene coding for the homeodomain is the T671C substitution—described several years ago—resulting in the Leu224Pro substitution in region III of the MSX1 homeodomain helix [29]. Predictive analyses point out that this mutation may completely inhibit the activity of the MSX1 protein, and its presence may result in congenital absence of second premolars and third molars in carriers [29].

As previously mentioned, mutations in the homeodomain encoding region of the *MSX1* gene associated with the occurrence of various forms of hypodontia, usually result in inhibition of the development of second premolars and third molars [30,31]. Other research teams also pointed out diverse effects of *PAX9* and *MSX1* gene variants on abnormalities in the development of specific types of teeth in humans. It was found that in the case of those two genes specific alleles may hinder or even stop the normal development of third molars. However, unlike *PAX9*, the presence of certain variants in the *MSX1* gene greatly impedes the development of second premolars [32,33]. It is, as some authors term it, “oligodontia of premolars” resulting from mutation in the *MSX1* gene, or “molar oligodontia” associated with defects in the *PAX9* gene [28]. It may result from the sequence of occurrences—described earlier in this chapter—that take place during tooth formation. In mouse embryos, in the early stages of tooth development, simultaneous expression of the *PAX9* and *MSX1* genes is observed, which means that the effects of mutations in any of these transcription factors may be compensated for [16], whereas in the successive stages of odontogenesis only *PAX9* expression is maintained at a sufficiently high level, so mutations in this gene mainly affect tooth primordia which develop at the end [34].

Bearing in mind the above-mentioned information on the impact of various mutations in the coding region of the homeodomain of the *MSX1* gene, it is interesting to note that the functional analyses of the five remaining missense mutations in the same region of the *MSX1* gene—that were correlated with hypodontia (Met61Lys, Ala194Val, Arg196Pro, Ala219Thr, and Ala221Glu)—revealed that none of the well-known molecular mechanisms, including disturbance of the interaction of MSX1 with PAX9 or another regulatory protein, reduction of the stability of the MSX1 protein, or reduction of the ability to bind to DNA of target genes, allowed to fully explain the importance of amino acid substitutions in the MSX1 protein in pathogenesis of hypodontia in humans. Moreover, it was demonstrated that, in reality, most of the aforementioned missense mutations in the *MSX1* gene do not impair the most important activity of the MSX1 protein, i.e., ability to interact with *PAX9* to activate expression of the *BMP4* gene, suggesting that the role of the MSX1 protein in tooth development abnormalities is indirectly related to interaction with *PAX9* [35]. It allowed to formulate a hypothesis that a search for potential molecular targets responsible for dental abnormalities should focus on other regions of the *MSX1* gene—not the regions coding for the homeodomain.

An example of promising variants of the *MSX1* gene—in the context of being relevant to the emergence of various abnormalities in tooth development—are polymorphic loci, *rs8670* and *rs12532*, located in the untranslated region of exon 2. At this point, it is worth explaining that with regard to human genes, the meaning of the term ‘exon’ is not only limited to the coding sequence, as many non-coding (fully or partially) exons have been described in human genes [36]. It is in this non-coding region of exon 2 of the *MSX1* gene that polymorphic variants, *rs8670* and *rs12532*—which were originally correlated with non-syndromic, sporadic oligodontia in humans—were described for a Polish population, [37]. For the first time, these results suggested that the non-coding regions of the *MSX1* gene, unrelated in any way to the direct functioning of the homeodomain, may also play a role in the etiology of tooth development abnormalities. Studies of the *rs8670* variant in Brazilian patients indicated a potential positive correlation of the occurrence of non-syndromic hypodontia of the upper lateral incisors in rare TT homozygotes, and their authors suggested that this polymorphic locus may reduce stability of the nascent mRNA, affect the *MSX1* gene via antisense RNA or it may disrupt translation processes [38]. However, other studies conducted on a larger group of Brazilian patients did not show statistically significant differences in the distribution of genotypes and alleles described at *rs8670* and *rs12532* between the group of patients with hypodontia and the control group with all teeth erupted [39].

The literature contains a small amount of research studies regarding *MSX1* gene expression and how it affects tooth impaction. Olsson et al. presented in their publication the effect of *MSX1* gene expression on the depth of maxillary third molar impaction. The study provided evidence that gene expression depends on the depth of tooth impaction. The polymorphisms presented by the authors are different to the polymorphisms in their own study. Unfortunately, the study was carried out on a small study group (*n* = 32) [40].

Another study by a team from India led by Devi determined the effect of *MSX1* polymorphism on palatal impaction of maxillary canines. It showed a statistically significant association between *rs12532* and palatal impaction of maxillary canines. Additionally, the presence of the AG/CT genotype indicated a significantly higher risk of palatal impaction. Unfortunately, the study was carried out only on a small group of Asian population. Gene polymorphisms were assessed only for palatal impaction of maxillary canines [41].

None of the studies assessed *MSX1* gene polymorphisms for impacted teeth in the mandible.

The *rs8670* and *rs12532 loci* in the *MSX1* gene were also analyzed in this experiment. The analyses showed that the frequencies of genotypes and alleles described at particular polymorphic loci in the *MSX1* gene did not differ significantly between the study group of patients with impacted teeth and the control group of patients with a properly erupted set of teeth. After the two subgroups of patients were distinguished in the study group—depending on the number of impacted teeth marked as NIT ≤ MED and NIT > MED—it was possible to reveal statistically significant differences. Statistically significant differences were found in the frequency of genotypes described at the *rs12532* polymorphic locus in the *MSX1* gene. AA genotype was much more common in people from the NIT > MED subgroup (66.2%) with impacted teeth whose number was higher than the median observed in this study, i.e., in people with more than three teeth impacted, compared to the subgroup of NIT ≤ MED subjects (46.8%) with a smaller number of impacted teeth. AA homozygotes were also more frequent in the NIT>MED group, compared to the control group (53.2%) who had no teeth impacted at all. However, the observed differences were not statistically significant. The frequency of the A allele at *rs12532* of the *MSX1* gene was significantly higher (f. A 0.8) in the subgroup of patients with a bigger number of impacted teeth (NIT > MED), compared to the subgroup in which only three or fewer impacted teeth were found (f. A 0.8), as well as compared to the patients from the control group with a complete set of erupted teeth (f. A 0.72). It may indicate that the presence of the A allele or the AA genotype described at *rs12532* in the *MSX1* gene is a genetic factor which is positively correlated with an increasing risk of the occurrence of the phenomenon of a small number of teeth impacted.

With regard to the analysis of haplotypes reconstructed on the basis of genotypic data described at polymorphic loci within the *MSX1* gene, three haplotypes of the *MSX1* gene [*rs8670*; *rs12532*] were identified in the present study: [C; A], [C; G], [T; A]. However, no statistically significant differences were found in the frequency of the haplotypes between the study group and the control group. Irrespective of the assumed haplotypic effect (additive, dominant, and recessive)—both at the level of global tests and the level of tests for significance conducted for particular haplotypes—no haplotypes in the *MSX1* gene described for the polymorphic loci under study showed association with belonging to the study group or the control group. On the other hand, the analyses of the relationship between the *MSX1* gene haplotypes and the number of impacted teeth revealed that at the level of global tests there is a relationship with the total number of impacted teeth in a particular patient. In the case of the *MSX1* gene, statistically significant differences in the frequency of the occurrence of haplotypes were found both for the additive and dominant effects assumed. At the level of individual tests for significance for the three haplotypes described at the *MSX1* gene, the [C;G] haplotype was significantly less frequent (f [C;G] 0.20) in the subgroup of NIT > MED patients with a total number of impacted teeth which was bigger than three (NIT > MED), compared to the NIT≤MED subgroup patients with a smaller number of improperly erupted teeth (f. [C;G] 0.32; *p* = 0.013 for the additive model, *p* = 0.008 for the dominant model). It is also worth noting that the same haplotype was more frequent in the control group of patients with teeth properly erupted (f. [C; G] 0.28). However, no differences exceed the level of statistical significance (*p* = 0.949 for the additive model, *p* = 0.808 for the dominant model). The above results may indicate a possibly protective nature of this arrangement of alleles described successively at the *rs8670* and *rs12532* loci of the *MSX1* gene in the context of a predisposition to the phenomenon of numerous teeth impacted in carriers of this arrangement of haplotypes.

## 4. Materials and Methods

### 4.1. Study Participants

Participation in the research project was offered to 392 patients consecutively reporting to the Department of Oral Surgery. The criteria, by which the patient was qualified for the study group, included presence of at least one tooth completely impacted in the maxillary or mandibular bone. The tooth, which was considered impacted, was surrounded by bone in the X-ray image and was absent in the dental arch at the time of eruption. The study group included 204 persons—82 males and 122 females—who were identified with one up to seven impacted teeth. Patients under sixteen, and people for whom it was impossible to get a panoramic radiograph, as well as those who had numerous missing teeth—which made it impossible to provide a diagnosis of hypodontia—were not qualified for the project. Relatives were also excluded from the project.

Based on the presence or absence of a diagnosis of tooth/teeth impaction, provided by a medical specialist, the study participants were divided into two groups:

(1) a study group which included patients with at least one permanent tooth impacted in the maxillary or mandibular bone as well as patients with supernumerary teeth.
(2) a control group including patients who had no impacted teeth in the maxillary bones and whose permanent teeth were emerged and set in the tooth arches correctly.

All patients participating in the study were informed about the purposefulness, benefits and the research method applied. After giving a written consent to participate in the project, they were provided with an individual number which made their identification impossible. The study began with collecting medical histories and physical examinations of the oral cavity. Then panoramic radiographs were taken. After physical examination of the oral cavity and analysis of the X-ray image, the patient was assigned to one of the two groups. The patients were qualified by two independent researchers (medical specialists in oral surgery). Each missing tooth that indicated hypodontia, an impacted tooth, a supernumerary tooth was marked on the diagram attached to the patient’s card.

The control group consisted of patients with no impacted tooth found during clinical and radiological examination. The control group included patients in whom no tooth impaction was found, with all permanent teeth erupted and correctly positioned in the dental arch. The participants were matched to the control group with a proper sex ratio, comparable to that observed in the study group. In total, the control group consisted of 188 people (81 males and 107 females) without any abnormalities in tooth eruption. Both in the study group and the control group two patients in the process of genetic testing provided the result of genetic testing which was unclear or was not recorded as a result of the absence of signal.

In order to mark teeth that were impacted, the two-digit Viohl system—officially adopted by the FDI Commission (French, Fédération Dentaire Internationale)—was used [42]. It is in line with the ISO system (International Standards Organization Designation System), also known as ISO 3950 (ISO 3950:2009 Dentistry—Designation system for teeth and areas of the oral cavity), and currently recommended by the World Health Organization (WHO).

All procedures employed throughout the study followed the guidelines of the World Medical Association’s Declaration of Helsinki [43] and were approved by the Bioethical Committee of the Medical University (Resolution No. KB-0012/39/16 of 25 April 2016). All procedures related to conducting genetic association analyses were designed and performed in compliance with the list of guidelines specified in the STREGA declaration (Strengthening the Reporting of Genetic Association Studies Statement [44]). All patients, who were pre-qualified to participate in the study, were informed about the project’s details, its aim, nature of their participation in the study as well as benefits and possible risks of participating in the study. Each potential study participant was informed in writing about the project, had time to read this information and the opportunity to have questions for the person who recruited him or her. Each patient was assured that both his or her consent to participate in the study, and refusal to do so, would not, in any way, affect his or her further treatment, and that his or her participation in the project would be anonymous and confidential. After learning the project’s details and making sure that all the items of information are clear, and after clarifying doubts by the recruiter, each patient, who decided to participate in the study, was asked to sign a declaration of informed consent to voluntarily take part in the study directly, or—in the case of participants under eighteen—with a legal guardian acting as proxy.

### 4.2. Biological Material Collection

Biological material from the patients qualified for the study group and control group was collected using epithelial mouth swabs in the Department of Oral Surgery. Prior to this, the participants were asked to rinse their oral cavity with distilled water, and then a swab was taken by vigorously rubbing epithelium of the inner part of the patient’s cheeks using sterile, nylon flocked microrheological swabs sealed in a test tube without substrate (FLOQSwabs COPAN, Brescia, Italy). In this way, epithelial cells were collected from the patient. Genetic material was isolated at a further stage of the study. The swab was inserted into the oral cavity in such a way that the tip of the applicator touched no anatomical structures. According to the manufacturer’s instructions, the tip was put to the mucous membrane of the cheek and was rubbed for 30 s in a circular and sliding motion until the applicator bristle turned light green. The tip was then placed in a sterile, sealed test tube. Each of them had its individual number identical with the patient’s number. The collected swabs were then placed at 8 ºC in two plastic containers—one for each group—in order to protect genetic material from degradation, and then transported to the laboratory where they had been stored deep frozen until the analyses were made.

### 4.3. Genotyping of Selected Polymorphic Loci

In order to detect selected polymorphisms in the genes under study, analyses were carried out following the method based on the polymerase chain reaction (PCR) derived from Real-Time PCR techniques using TaqMan probes. The method is based on the 5′–3′ exonuclease activity of Taq polymerase to degrade complexes of dual-labelled fluorescent probes hybridized with complementary sequences in target genes. Taq probes have a fluorescence reporter at the 5′ end (e.g., FAM, NED), and a fluorescence quenching molecule (e.g., TAMRA, DABCYL) at the 3′ end. The size of the probe (appropriate distance between the reporter at one end and the quencher at the other) allows to transfer energy between fluorochromes (fluorescence resonance energy transfer—FRET), and this, as a result, inhibits emission of a recordable fluorescence signal. After binding to a complementary sequence within the selected molecular target, at a stage of PCR extension the probe is degraded by Taq polymerase with the 5′ exonuclease activity. As a result, fluorochrome is separated from the quencher, and separation of both molecules enables fluorescent light emission [45].

The genotyping procedure itself was the same for all polymorphic loci under study and was carried out using reaction mixtures of 10 µL per sample. Each sample was genotyped in duplicate. Apart from DNA isolated from a particular sample, the mixture included the following reagents: sterile water, reaction buffer with Mg^2+^ (10×): 700 mM Tris-HCl pH 8.6/25 °C, 166 mM (NH4)_2_SO_4_, 25 mM MgCl_2_ (Novazym, Poznań, Poland), an aqueous solution of deoxyribonucleoside triphosphates—dATP, dCTP, dGTP, and dTTP—with a concentration of 2.5 mM each (Novazym, Poland), thermostable polymerase with the 5′–3′ exonuclease activity (AllegroTaq DNA Polymerase 5 U/µL, Novazym, Poland) and the TaqMan Pre-Designed SNP Genotyping Assays (20×) kit containing a couple of primers and two TaqMan molecular probes binding to the minor groove of DNA (Minor Groove Binding—MGB). Each probe was complementary with a specific allelic version that could occur at a polymorphic locus under study and was labelled with the FAM dye emitting a fluorescent signal after laser excitation in the green light color range or the VIC dye emitting a fluorescent signal after laser excitation in the yellow light color range (Life Technologies, Carlsbad, CA, USA). When for a particular sample a fluorescence signal was observed only on the green channel (Green 8), it was classified as a genotype of homozygote characteristic for the first type of allele at a particular polymorphic locus. When fluorescence emission was found only on the yellow channel (Yellow 10), the sample was assigned a genotype of homozygote characteristic for the second type allele. When the signal was observed on both channels simultaneously, a heterozygous genotype was described in the sample.

The details of the TaqMan Pre-Designed SNP Genotyping Assays kits used for genotyping specific polymorphic loci in the *MSX1* gene are presented below.

Detection of genotypes in polymorphic loci in the *MSX1* gene.

- SNP C→T: single-nucleotide polymorphism within the UTR region of exon 2 (rs8670) was detected with Real-Time PCR using the TaqMan Pre-Designed SNP Genotyping Assay kit no. C__11357234_10 (Applied Biosystems, Waltham, MA, USA)
- SNP A→G: single-nucleotide polymorphism within the UTR region of exon 2 (rs12532) was detected with Real-Time PCR using the TaqMan Pre-Designed SNP Genotyping Assay kit no. C__26933394_10 (Applied Biosystems, Waltham, MA, USA)

The samples containing DNA isolated from the study group and control group patients and prepared reaction mixtures were placed in the C1000 Touch Thermal Cycler (Bio-Rad, Feldkirchen, Germany) and subjected to temperature which changed according to the applied thermal-time profile. The profile was the same for all reactions and was as follows:

- pre-denaturation at 95 °C for 5 min;
- 45 cycles including two stages:
- denaturation at 92 °C for 15 s,
- primer annealing and elongation at 60 °C for 1 min.

Increases in product quantity were recorded for each cycle after completion of the elongation stage. When, for a particular sample, a fluorescence signal was observed only on the green channel (Green 8), it was classified as a genotype of the first type of homozygote. When the emission of fluorescence was found only on the yellow channel (Yellow 10), the sample was described as a genotype of the second type of homozygote. When the signal was observed on both channels simultaneously, the sample was determined as a heterozygous genotype. Genotypes at all polymorphic loci under study were read off using the allele discrimination analysis in the Bio-Rad CFX Manager 3.1 software (Bio-Rad, Germany).

### 4.4. Statistical Analysis

Allele frequencies were determined based on genotype frequencies. The consistency of genotype frequencies with the theoretical Hardy–Weinberg distribution was tested using the Chi-square test. Genotype and allele frequencies were also compared between the groups using the Chi-square test. The haplotype analysis was performed using the haplo.stats suite (version 1.7.7) and the R statistical computing environment (https://cran.r-project.org, version 3.1.0, access date 5 April 2017) using the haplo.score function, with three models of haplotype inheritance assumed, i.e., additive (0 vs. 1 vs. 2 copies of a particular haplotype), dominant (homozygotes and heterozygotes for a particular haplotype exert the same effect) and recessive (only homozygotes for a particular haplotype are related to the trSIT under study). Haplotypes with a frequency of less than 5% were excluded in the analysis of association. The remaining statistical tests were performed using STATISTICA, version 12. The value *p* < 0.05 was adopted as the level of significance.

## 5. Conclusions

After the two subgroups of patients were distinguished in the study group based on the number of impacted teeth, statistically significant differences in the frequency of genotypes described for *rs12532* in the *MSX1* gene were found: the occurrence of the AA genotype was more distinct in people from the NIT>MED subgroup who had more than three teeth impacted, compared to the NIT≤MED subgroup with a smaller number of impacted teeth (66.2% vs. 46.8%, *p* = 0.026). The frequency of the A allele at *rs12532* of the *MSX1* gene was significantly higher in the subgroup of patients with a bigger number of impacted teeth, compared to the subgroup in which only three or fewer impacted teeth were found (f. A 0.8 vs. 0.68, *p* = 0.009).

## Figures and Tables

**Table 1:** Number of impacted teeth in study group patients.

Number of Impacted Teeth	N	% (Teeth) *n* = 554
1	44	21.6%
2	55	27.0%
3	28	13.7%
4	72	35.3%
5	3	1.5%
6	1	0.5%
7	1	0.5%

**Table 2:** Proportion of different tooth types (according to the Viohl system) in study group patients.

Viohl System	N	% (Teeth) *n* = 554	% (Patients) *n* = 204	Median
13	6	1.08%	2.94%	3
14	1	0.18%	0.49%	
17	2	0.36%	0.98%	Quartile 1
18	107	19.31%	52.45%	1
19	1	0.18%	0.49%	
23	11	1.99%	5.39%	
24	1	0.18%	0.49%	Quartile 3
27	1	0.18%	0.49%	11
28	111	20.04%	54.41%	
34	4	0.72%	1.96%	
35	3	0.54%	1.47%	Inter-quartile
38	154	27.80%	75.49%	10
43	1	0.18%	0.49%	
44	4	0.72%	1.96%	
45	1	0.18%	0.49%	Average
47	1	0.18%	0.49%	29.35
48	144	25.99%	70.59%	
49	1	0.18%	0.49%	

**Table 3:** Proportion of different tooth types (according to the Viohl system) depending on the number of impacted teeth in study group patients.

Viohl System	Study Group *n* = 204	TNIT 1 *n* = 44	TNIT 2 *n* = 55	TNIT 3 *n* = 28	TNIT 4 *n* = 72	TNIT 5 *n* = 3	TNIT 6 *n* = 1	TNIT 7 *n* = 1
18	107 (52.5%)	7 (15.9%)	6 (10.9%)	18 (64.3%)	72 (100%)	3 (100%)	1 (100%)	0
28	111 (54.4%)	6 (13.6%)	11 (20.0%)	18 (64.3%)	72 (100%)	3 (100%)	1 (100%)	0
38	154 (75.5%)	10 (22.7%)	45 (81.8%)	23 (82.1%)	72 (100%)	3 (100%)	1 (100%)	0
48	144 (70.6%)	7 (15.9%)	41 (74.5%)	21 (75.0%)	71 (98.6%)	3 (100%)	1 (100%)	0
13	6 (2.9%)	3 (6.8%)	1 (1.8%)	1 (3.6%)	0	1 (33.3%)	0	0
23	11 (5.4%)	6 (13.6%)	1 (1.8%)	2 (7.1%)	1 (1.4%)	1 (33.3%)	0	0
33	0	0	0	0	0	0	0	0
43	1 (0.49%)	1 (2.3%)	0	0	0	0	0	0
SIT	1/1/1/1/1/1/1 2/2/2 7	1/1/1/1	1 2/2	1	0	1	2	7

SIT—supernumerary impacted teeth, TNIT—total number of impacted teeth.

**Table 4:** Hardy–Weinberg’s law for the study and control group.

Hardy–Weinberg Equilibrium Calculator Including Analysis for Ascertainment Bias		Observed (Expected)		Test χ^2^	
				χ^2^	*p*
*MSX1 rs8670* Study group *n* = 202	C/C	113 (111.4)	C allele freq = 0.74 T allele freq = 0.26	0.352	>0.05
	C/T	74 (77.2)			
	T/T	15 (13.4)			
*MSX1 rs8670* Control group *n* = 186	C/C	115 (110.7)	C allele freq = 0.76 T allele freq = 0.24	3.037	<0.10
	C/T	60 (68.7)			
	T/T	11 (10.7)			
*MSX1 rs12532* Study group *n* = 203	AA	110 (106.4)	A allele freq = 0.72 G allele freq = 0.28	1.557	>0.05
	A/G	74 (81.1)			
	G/G	19 (15.4)			
*MSX1 rs12532* Control group *n* = 186	AA	99 (97.3)	A allele freq = 0.72 G allele freq = 0.28	0.406	>0.05
	A/G	71 (74.5)			
	G/G	16 (14.3)			

*p*-statistical significance χ^2^ test.

**Table 5:** Frequency of genotypes and alleles of the *MSX1 rs8670* and *rs12532* gene polymorphism in the study and control group.

	Freq. Genotypes			Freq. Alleles	
***MSX1 rs8670***	C/C *n* (%)	C/T *n* (%)	T/T *n* (%)	C *n* (%)	T *n* (%)
Study group *n* = 202	113 (0.56)	74 (0.37)	15 (0.07)	300 (0.74)	104 (0.26)
Control group *n* = 186	115 (0.62)	60 (0.32)	11 (0.06)	290 (0.78)	82 (0.22)
χ^2^ (*p*) OR [95% CI]	1.44 (0.487) OR C/C:C/T = 0.80 [0.52–1.22]; OR CC:T/T = 0.72 [0.32–1.64]; OR C/T:T/T = 0.90 [0.39–2.11]			1.46 (0.228) OR C:T = 0.82 [0.59–1.14]	
***MSX1 rs12532***	A/A *n* (%)	A/G *n* (%)	G/G *n* (%)	A *n* (%)	G *n* (%)
Study group *n* = 203	110 (0.54)	74 (0.37)	19 (0.09)	294 (0.72)	112 (0.28)
Control group *n* = 186	99 (0.53)	71 (0.38)	16 (0.09)	269 (0.72)	103 (0.28)
χ^2^ (*p*) OR [95% CI]	0.16 (0.925) OR A/A:A/G = 1.07 [0.70–1.63]; OR A/A:G/G = 0.95 [0.46–1.92]; OR A/G:G/G = 0.88 [0.42–1.84]			0.001 (0.975) OR A:G = 1.01 [0.73–1.38]	

*p*-statistical significance χ^2^ test, OR—Odds Ratio, significant OR (*p* < 0.05).

**Table 6:** Comparison of *MSX1* haplotypes between the study (Stu) and control (Con) group with statistical significance of effects (additive, dominant, and recessive); total number of haplotypes taken into account during analyses: *n* = 388.

*MSX1* NM_002448.3:c.[6C > T; 276A > G]		Frequency			Additive		Dominant		Recessive	
					1.56; df = 2; *p* = 0.458 (0.452) †		1.89; df = 3; *p* = 0.595 (0.568) †		0.65; df = 3; *p* = 0.885 (0.902) †	
*rs8670*	*rs12532*	General	Con	Stu	Score	*p*_a_	Score	*p*_a_	Score	*p*_a_
C	A	0.48	0.50	0.47	−0.97	0.331 (0.340)	−1.01	0.314 (0.306)	−0.57	0.569 (0.598)
C	G	0.28	0.28	0.27	−0.06	0.949 (0.916)	−0.24	0.808 (0.783)	0.28	0.782 (0.811)
T	A	0.24	0.22	0.26	1.18	0.240 (0.230)	1.18	0.239 (0.222)	0.59	0.552 (0.566)

*p*_a_—values calculated for haplotype frequencies, comparisons between the control and study group, minimal haplotypes’ frequency taken into account during analyses—5%, *p* values corrected according to the sex are given in parentheses, score—magnitude and direction of effect, †—global significance test.

**Table 7:** Genotypes and alleles described for *MSX1 rs8670* and *rs12532* variants in the subgroups of patients with a total number of impacted teeth below (TNIT ≤ MED) and above (TNIT > MED) median value.

	Freq. Genotypes			Freq. Alleles	
***MSX1 rs8670***	C/C *n* (%)	C/T *n* (%)	T/T *n* (%)	C *n* (%)	T *n* (%)
TNIT ≤ MED *n* = 125	76 (0.61)	40 (0.32)	9 (0.07)	192 (0.77)	58 (0.23)
TNIT > MED *n* = 77	37 (0.48)	34 (0.44)	6 (0.08)	108 (0.70)	46 (0.30)
χ^2^ (*p*) OR [95% CI]	3.33 (0.189) OR C/C:C/T = 1.74 [0.96–3.19]; OR C/C:T/T = 1.37 [0.45–4.14]; OR C/T:T/T = 0.78 [0.25–2.43]			2.22 0.136 OR C:T= 1.41 [0.90–2.22]	
***MSX1 rs12532***	A/A *n* (%)	A/G *n* (%)	G/G *n* (%)	A *n* (%)	G *n* (%)
TNIT ≤ MED *n* = 126	59 (0.47)	53 (0.42)	14 (0.11)	171 (0.68)	81 (0.32)
TNIT > MED *n* = 77	51 (0.66)	21 (0.27)	5 (0.07)	123 (0.80)	31 (0.20)
χ^2^ (*p*) OR [95% CI]	7.28 (0.026 *) OR A/A:A/G = 0.46 [0.24–0.86] #; OR A/A:G/G = 0.41 [0.14–1.23]; OR A/G:G/G = 0.90 [0.29–2.82]			6.91 (0.009 *) OR A:G = 0.53 [0.33–0.85] #	

*p*-statistical significance χ^2^ test, * significant statistical differences OR—Odds Ratio, # significant OR (*p* < 0.05).

**Table 8:** Association analysis between *MSX1* (*rs8670*; *rs12532*) haplotypes and total number of impacted teeth; total number of haplotypes taken into account during analyses: *n* = 202.

*MSX1* NM_002448.3:c.[6C > T; 276A > G]		Frequency			Additive		Dominanat		Recessive	
					6.44; df = 2; *p* = 0.040 * (0.038 *) †		9.65; df = 3; *p* = 0.022 * (0.021 *) †		1.30; df = 3; *p* = 0.729 (0.720) †	
rs8670	rs12532	General	TNIT ≤ MED	TNIT > MED	Score	*p*	Score	*p*	Score	*p*
C	G	0.27	0.32	0.20	−2.48	0.013 * (0.012 *)	−2.64	0.008 * (0.008 *)	−1.11	0.266 (0.254)
C	A	0.47	0.45	0.50	1.01	0.313 (0.297)	1.64	0.102 (0.100)	−0.05	0.957 (0.987)
T	A	0.26	0.23	0.30	1.46	0.144 (0.147)	1.77	0.076 (0.075)	0.16	0.876 (0.899)

*p*—values calculated for haplotype frequencies, comparisons between the subgroup of patients with a total number of impacted teeth below the median value (TNIT ≤ MED) and the subgroup of patients with a total number of impacted teeth above the median value (TNIT > MED), minimal haplotypes’ frequency taken into account during analyses—5%, values corrected according to the sex are given in parentheses, score—magnitude and direction of effect, †—global significance test, * statistical significant difference *p* < 0.05.

## Data Availability

Data available on request due to restrictions—privacy or ethical.
